# Tunable Clamped–Guided Arch Resonators Using Electrostatically Induced Axial Loads

**DOI:** 10.3390/mi8010014

**Published:** 2017-01-04

**Authors:** Nouha Alcheikh, Abdallah Ramini, Md Abdullah Al Hafiz, Mohammad I. Younis

**Affiliations:** 1 Physical Science and Engineering Division, King Abdullah University of Science and Technology (KAUST), Thuwal 23955-6900, Saudi Arabia; nouha.alcheikh@kaust.edu.sa (N.A.); abdallah.ramimi@kaust.edu.sa (A.R.); 2 Computer, Electrical and Mathematical Science and Engineering Division, King Abdullah University of Science and Technology (KAUST), Thuwal 23955-6900, Saudi Arabia; abdullah.hafiz@kaust.edu.sa

**Keywords:** microresonators, clamped–guided arch beams, bi-directional electrostatic actuator

## Abstract

We present a simulation and experimental investigation of bi-directional tunable in-plane clamped–guided arch microbeam resonators. Tensile and compressive axial forces are generated from a bi-directional electrostatic actuator, which modulates the microbeam stiffness, and hence changes its natural frequency to lower or higher values from its as-fabricated value. Several devices of various anchor designs and geometries are fabricated. We found that for the fabricated shallow arches, the effect of the curvature of the arch is less important compared to the induced axial stress from the axial load. We have shown that the first mode resonance frequency can be increased up to twice its initial value. Additionally, the third mode resonance frequency can be increased up to 30% of its initial value. These results can be promising as a proof-of-concept for the realization of wide-range tunable microresonators. The experimental results have been compared to finite-element simulations, showing good agreement among them.

## 1. Introduction

Microelectromechanical systems (MEMS) resonators have received significant attention in the science community due to their advantages, such as small size, high sensitivity, high resolution, low power consumption, and high quality factor [1,2,3]. MEMS resonators can be employed in different applications, such as bio/chemical sensors [4], viscosity sensors [5], oscillators and filters [6,7], energy harvesters [8,9], and logic gaits [10].

In many applications (such as wireless communications), it is desirable to have an active mechanism to tune the resonance frequency of oscillators and filters while in use. This in-demand tuning can be a desirable feature to minimize noise and signal interference. It also expands the range of use of a single resonator to various applications. Additionally, the resonance frequency of a MEMS resonator can deviate from its desired value due to many factors, such as residual stresses, fabrication imperfections, and changes in temperature and pressure. To compensate these effects, post-fabrication tuning mechanisms are highly desirable.

The resonance frequency depends principally on the geometry, structure stiffness, material properties, and the stresses induced from the fabrication process and external loads. The various geometrical and anchoring properties of the structures—such as cantilevers, clamped–clamped beams, and plates—change the resonant frequency [11,12,13,14,15]. Many materials have been used for microresonators, such as Silicon and SiC [16,17]. In mass sensing applications, micromechanical resonators are sensitive to the small added mass. Therefore, due to the added mass on the structure, the resonant frequency can be shifted [18,19].The maximum sensitivity is attained when the resonator is operated exactly at the resonance peak [20]. Therefore, it is desirable to have the ability to actively tune the natural frequency of the structure to ensure that it is operated at the optimal operating conditions.

Different methods have been investigated to change the resonant frequency of resonators, such as magnetic [10], thermal [20,21,22,23], and electrostatic [1,24,25,26]. Zhu et al. [27] used the electromagnetic effective tuning on a cantilever realized by applying an axial tensile load using a pair of tuning magnets. The resonant frequency was tuned from 67.6 to 98 Hz by changing the distance between two magnets. Hajjaj et al. [28] utilized an electrothermal scheme to tune the resonant frequency of a clamped–clamped microbeam resonator. They showed that the resonant frequency can be increased up to twice its initial value. Pourkamali et al. [29] designed self-aligned vertical capacitive gaps for electrostatic tuning. The resonant frequency was tuned from 505 to 450 kHz. Kosinsky et al. [11] used electrostatic tuning for out-of-plane and in-plane clamped–clamped beams. They demonstrated the ability to tune the resonant frequency of these beams bi-directionally.

In recent years, the static and dynamic behaviors of arch microbeams under electrostatic forces have been explored [30,31,32]. However, few works have investigated the effect of the axial forces for such structures. Alkharabsheh et al. [33] investigated the effect of axial forces on the static behavior and the natural frequency of electrostatically actuated arches using the Galerkin procedure. Elata et al. [34] experimentally and theoretically studied the electromechanical buckling response under bi-directional axial loads. Krylov et al. [35] examined the two-directional switching of curved pre-buckled electrostatically actuated beams under axial loads. However, high tuning of the beams under bidirectional axial force using electrostatic actuation has yet to be investigated in the literature. Many MEMS applications still need high tuning frequency as filters. Additionally, the ability to tune the resonance frequency in both directions (increasing and decreasing) is rare. This study represents a proof of concept for a mechanism for tuning devices through axial loads, which can be used in several applications, such as filters, logic devices, and resonators. Further, this mechanism can provide parametric excitation, which has many practical applications in MEMS [36].

In this paper, we demonstrate highly tunable in-plane clamped–guided shallow arch beams using axial loads generated from electrostatic forces. The devices are designed and simulated using a finite-element model of the software COMSOL (COMSOL, Inc., Burlington, MA, USA). Measurements are performed for various values of tensile and compressive axial loads. Comparison between the simulation and experimental results are presented, showing good agreement among the results.

## 2. Design and Principle of the Tunable Microresonators

**Figure 1:**
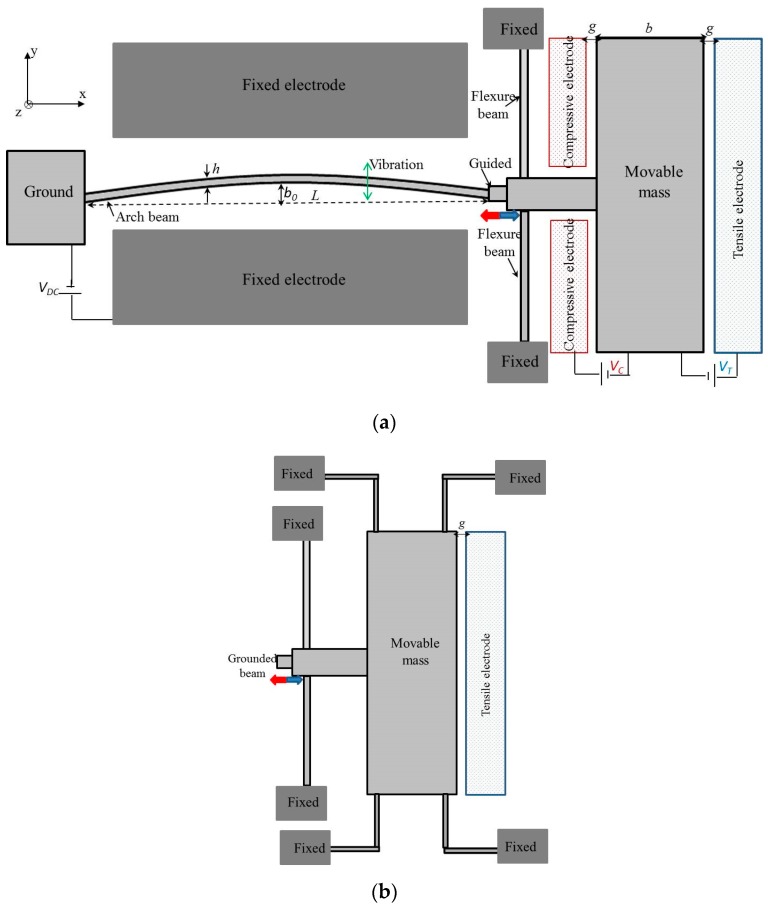
Schematic of the resonators. (**a**) Type 1: a fixed-guided microbeam is suspended by two flexure beams with a large mass and electrostatic actuation electrodes for compressive or tensile axial force; (**b**) Type 2: the mass is suspended by four flexure beams.

As shown in Figure 1, resonators with a clamped–guided arch microbeam of a half-wave sine type are designed with electrostatic actuators for the application of tensile and compressive axial loads. To avoid the high rotation stiffness of the structure in the *y*–*z* plane, the guided structure is suspended with two flexure beams. The dimensions of the flexure beams were chosen as 460 and 5 µm for their length and width, respectively. For these parameters and the parameters of the arch beam (Sample “A” in Table 1), the stiffness ratio is approximately 0.0014, and then the tensile and compressive axial loads are applied directly on the arch beams. One end of the arch beam is anchored, while the other end is connected to a large mass (*m*). The proposed design allows bi-directional movement of the arch beam in the *x*-axis. When an actuation voltage is applied between the tensile (compressive) electrode and the mass, an axial tensile (compressive) stress is generated within the beam. The clamped–guided arch beam is between two parallel electrodes (Figure 1a). To induce the vibration, it is excited electrostatically by a DC polarization voltage (*V* = 40 V). As shown in Figure 1, two-different architectures were designed for the experimental work. Type 1 (Figure 1a) comprises a movable mass guided by two flexural beams with both tensile and compressive electrodes. Type 2 (Figure 1b) consists of a movable mass supported by four L-shaped flexure beams with only a tensile electrode.

**Figure 2:**
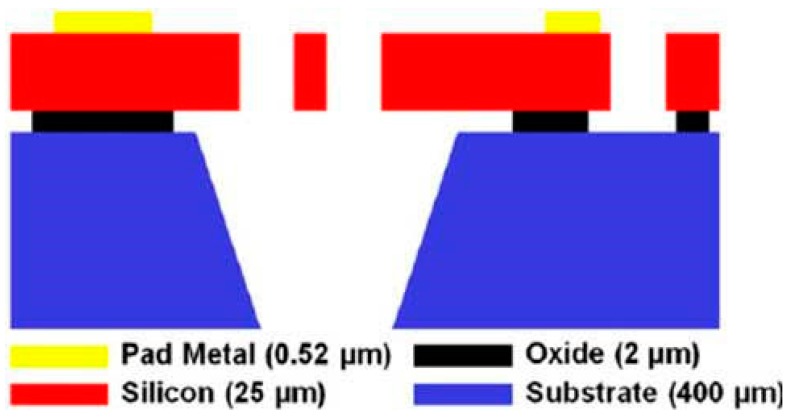
Cross-sectional view of the fabricated structure.

We used commercial foundry process SOIMUMPs by MEMSCAP (MEMSCAP Inc., Durham, NC, USA) [37] to fabricate our devices. Figure 2 shows a cross-sectional view of a SOIMUMPs processed device. For the detailed fabrication process, please see [37]. In brief, the process begins with a 150 mm silicon on insulator (SOI) wafer. The top surface of the silicon device layer is doped by depositing a phosphosilicate glass (PSG) layer and annealed at a high temperature. Then, a metal stack of 20 nm of chrome and 500 nm of gold is deposited using the patterns in the first mask. Then, the silicon device layer is lithographically patterned using the second mask and etched using deep reactive ion etching (DRIE). This step defines the device structure. Next, a protective material is applied to the top surface of the silicon layer to protect it from any damage due to subsequent processing. The substrate layer is lithographically patterned from the bottom surface using the third mask. The bottom oxide layer is removed using RIE, and a DRIE etch is used to remove the substrate completely in this area. Then, the buried oxide is removed using a wet etch. Next, the protective material is stripped in a dry etch process. In this step, the mechanical structures in the silicon layer are released. The remaining oxide layer is removed using a vapor HF process.

**Figure 3:**
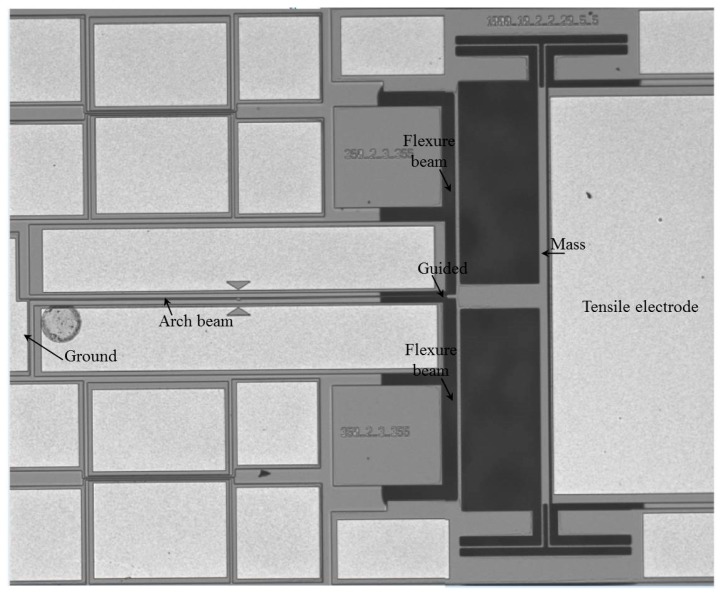
A photo of the fabricated resonator of Type 2 for an arch beam of *L* = 1000 μm.

The dimensions of the devices and fabricated curved beams are listed in Table 1 for Type 1 and Type 2 designs. The beams were 600 and 1000 μm in length (*L*), 1.85 μm in width (*h*), and 25 μm (Si device layer of SOI wafer) in thickness. The gap between the actuating electrode and the resonating beams was 8 μm at the clamped ends and either 10.6 μm or 9.8 μm at the mid-point of the microbeam, due to the initial curvatures (*b*_0_). The two flexure beams—which are used to guide the microbeams—had length of 460 μm and width of 10 μm. The width of the four folded flexure beams suspended to the mass of Type 2 was 2.5 μm. Figure 3 shows a picture of one of the fabricated resonators of Type 2. Note that the handle and box layer has been totally removed in some selected areas of the structure of Type 2; hence in Figure 3, those areas show black color due to poor reflection of light. Note also that the focus for Type 2 structure resonance frequency tuning was by means of axial tensile stress, hence the compressive electrode was intentionally removed.

## 3. Finite Element Model

**Figure 4:**
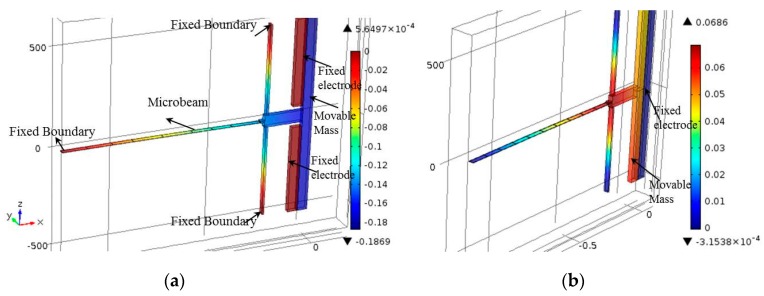
Finite element model (FEM) static simulation results of the *x*–displacement of the Type 1 actuator with *L* = 1000 μm, *h* = 1.8 μm, *b*_0_ = 1.8 μm, *b* = 50 μm, and *g* = 5 μm under (**a**) a compressive load of *V*_C_ = 50 V and (**b**) a tensile load of *V*_T_ = 40 V.

A finite element (FE) model was built in COMSOL to design the devices and to simulate their responses after fabrication. To account for the various physical domains of the problem, the electromechanics solver was utilized, which accounts for both the electrostatic and mechanical domains. By solving the stationary problem of the structure, the in-plane displacement can be found. By adding the eigenfrequency study to the stationary one, the natural frequencies of the devices can be calculated. Polysilicon was used as the material in the simulation. The structure consists of the movable part and fixed part, as shown in Figure 4a. The movable part includes the arch beams, two flexure beams, and the large mass, while a fixed part was used in the electrodes (compressive and tensile electrodes). All the movable parts were connected to the ground, and the fixed electrode was connected to a voltage source. Tetrahedral elements were used as the element type to mesh the structure when the meshing size was set to be finer. Here, we focus on the modes that resemble the first and third modes of a shallow arch.

**Figure 5:**
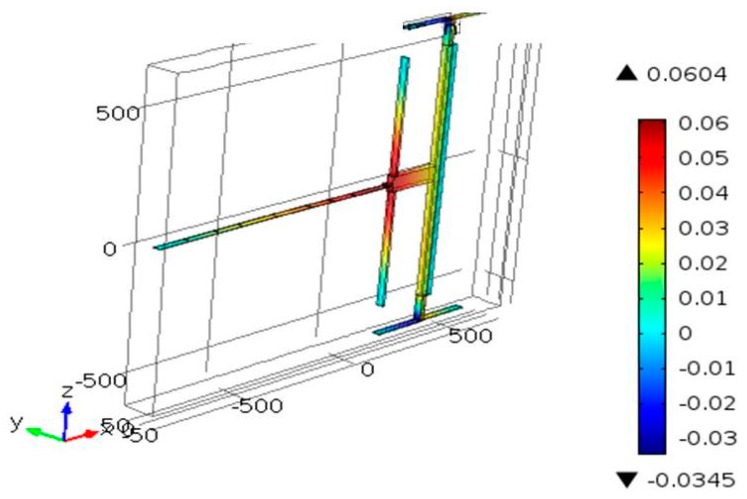
FEM static simulation results of the *x*–displacement of the Type 2 actuator with *L* = 1000 μm, *h* = 1.8 μm, *b*_0_ = 1.8 μm, *b* = 10 μm, and *g* = 2 μm under a tensile load of *V*_T_ = 40 V.

Figure 4a,b show the bidirectional *x*-displacement motion of the actuator, as obtained from the FE model, for a compressive and tensile load, respectively. As shown in Figure 4b, the design of Type 1 does not have symmetric in-plane *x*-displacement along the tensile direction, allowing the rotation of the large mass (rotation about the axes) even at the presence of two flexural support beams. The design of Type 2, with four-L shaped beams, on the other hand, shows symmetric *x*-displacement along the tensile direction (Figure 5).

## 4. Results and Discussion

**Figure 6:**
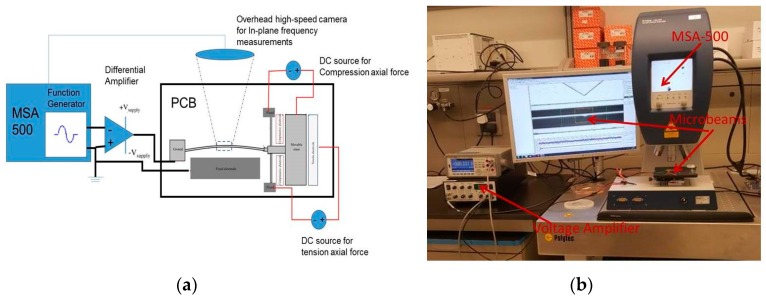
Experimental setup: (**a**) schematic and (**b**) photograph of setup. MSA: Micro System Analyzer.

To experimentally characterize the devices, we utilized a Micro System Analyzer (MSA-500, polytec Inc., Irvine, CA, USA) with in-plane dynamic measurement system using a stroboscopic video microscopy from Polytec [38] (Figure 6). We used the ring down measurement and the fast Fourier transform (FFT) to measure the resonance frequencies of the clamped–guided beams for each value of *V*_C_ and *V*_T_, applied between the large mass and the corresponding compressive/tensile electrodes. In this measurement method, we applied a sudden impulse of an electrostatic load (*V*_DC_) on the beam and the actuation electrode (fixed electrode), allowing the beam to vibrate freely (ring down) until the motion dies out. To amplify the generated *V*_DC_ voltage by the Micro System Analyzer (MSA), an amplifier was used, and then the voltage was applied between the beam and the fixed electrode.

**Figure 7:**
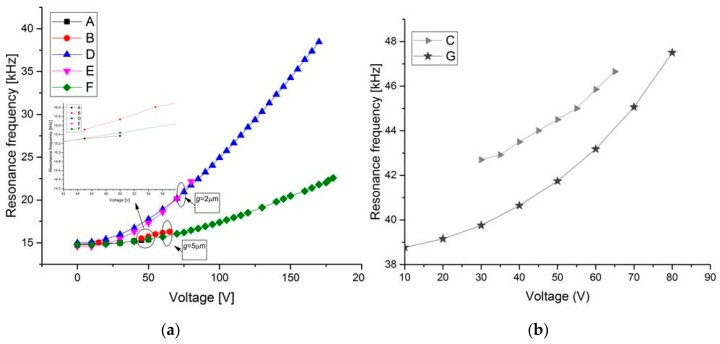
Measured change in resonant frequency with tensile axial load for various designs in Table 1 of Type 1 and Type 2 for the first resonant mode. (**a**) *L* = 1000 μm; (**b**) *L* = 600 μm.

All the structures of Table 1 were tested. Figure 5a,b show the measurement results of the resonant frequency for the first mode of several beams of Type 1 and Type 2 with varying tensile axial load. It shows that the resonant frequency of the beam increases when the electrostatic voltage increases, and hence the tension inside the arch increases. For the third mode, it can be observed from Figure 7 that the resonant frequency increases. One can note here that for an axial tensile load, the frequency tuning is governed by two mechanisms: the decrease in the stiffness of the arch due to the decrease in its arch rise (*b*_0_) (which decreases the resonance frequencies), and the increase in the axial stress due to the axial tensile load (which increases the resonance frequency). However, the results in Figure 5 and Figure 6 show that—for the current designs—the axial stress effect dominates the reduction in stiffness due to the decrease in curvature, and thus results in increasing the resonant frequency. This dominant effect can be attributed to the low initial curvature of the arches compared to their length.

**Figure 8:**
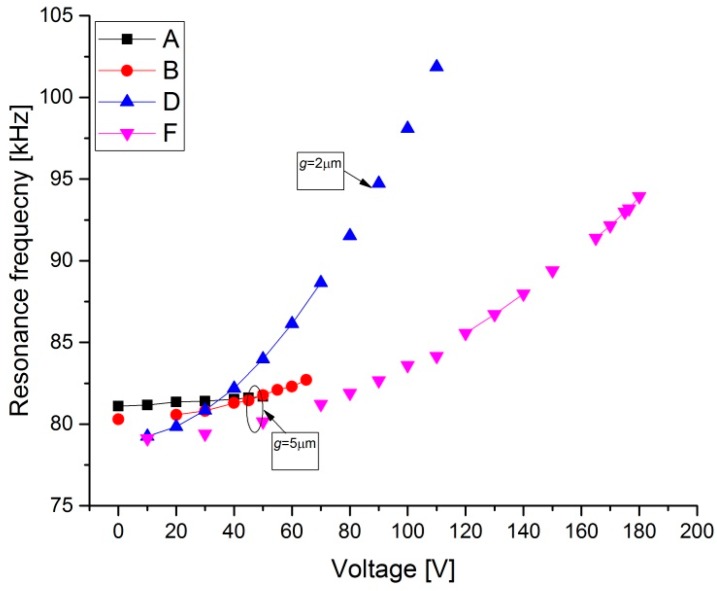
Measured change in resonance frequency with tensile axial load for various designs in Table 1 for Type 1 and Type 2 for the third mode of the 1000 μm length beams.

As shown in Figure 7a, for structures A and B, with a gap *g* = 5 μm, a maximum tuning voltage of 55 and 65 V, respectively, is required for resonant frequency tuning from 14.74 to 15.37 kHz for structure A and from 14.87 to 16.3 kHz for structure B. In this case, the maximum percentage change in the resonance frequency is estimated to be 4% and 10%, respectively (Δ*fr* = (*fr* [*V*_DC_] − *f*_0_ [*V*_DC_ = 0]) × 100/*f*_0_). For the third mode (Figure 8), the results show relatively less resonant frequency shift (3%). For structure D, Figure 7a shows a remarkable increase in the first mode resonant frequency from 15 to 39 kHz (160%). For the third mode (Figure 8), it increases from 79 to 102 kHz (29%) for a maximum tuning voltage of 110 V. With the arch beams length of 600 μm (Figure 7b), the first mode resonant frequency can be tuned from 42 to 46.65 kHz (11%) for structure C by increasing the electrostatic voltage up to 65 V. For structure G, the tuning range is about (23%) from 38.7 to 47.5 kHz for 80 V. It can be inferred that for type 2 (D and G) design, the resonant frequency can be tuned more than Type 1 (A, B, and C) design, both for the first and third mode.

**Figure 9:**
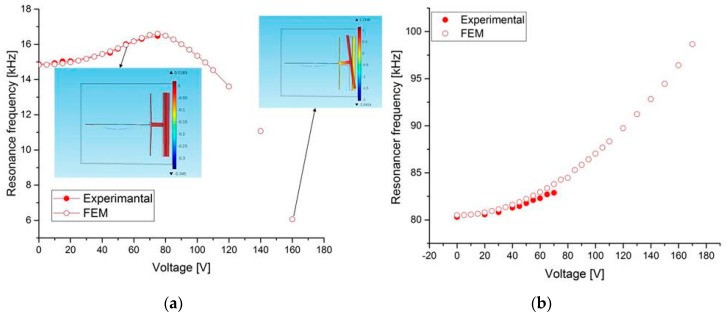
Measurements and FE simulations of the resonant frequency tuning with the tensile axial load for structure B of Type 1 at (**a**) first mode and (**b**) third mode.

**Figure 10:**
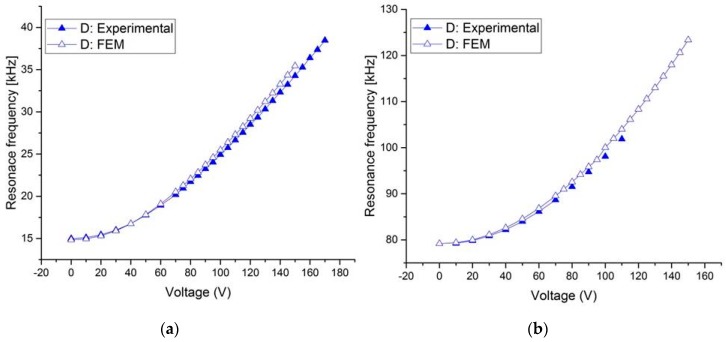
Measurements and FE simulations of the resonant frequency tuning with the tensile axial load for structure D of Type 2 at (**a**) first mode and (**b**) third mode.

To further understand the limit of the resonant frequency tuning capability of Type 1 design, the experimental results of structure B are compared with the FE results. As shown in Figure 9a, the resonant frequency of the first mode initially increases up to 75 V, and after that it decreases down to 6 kHz at 160 V. For the third mode (Figure 9b), it increases from 80 to 98 kHz (23%) for a maximum tuning voltage of 170 V. This is due to the effect of the rotation of the large mass on the beam at 75 V and higher voltage loads. Thus, for Type 1, as the simulation result shows, the rotated mode appears after 75 V, and then the large mass collapses with one of the two electrodes (tensile and compressive electrodes). On the other hand, for Type 2 (Figure 10), this effect is almost negligible. A wide tunability is shown for the first (Figure 10a) and the third modes (Figure 10b). This tunability depends on the stiffness of the mass and the four L-shaped beams. Although the maximum shown applied voltage is 170 V, due to the limitation in the power source in principle, more tunability can be achieved at higher voltage load. This wide resonant frequency tuning is primarily attributed to the *x*-displacement with large electrostatic voltages. Thus, for this design (Type 2), we can obtain more tunability with less voltage actuation for different stiffness guided arch beams.

**Figure 11:**
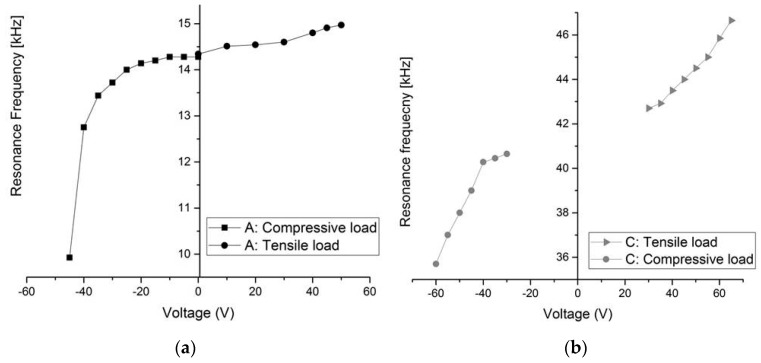
Measurements of the change in resonant frequency of the first mode with bi-directional axial loads for Type 1. (**a**) Structure A; (**b**) Structure C.

Figure 11 shows the resonant frequency tuning results of the first mode obtained by varying the axial loads for structures A and C (Type 1). This shows that a compressive load has more effect in this case on the resonant frequency compared to the tensile load. This figure shows that an axial tensile load applied to the arch clamped–guided beams increases the resonant frequency of the beams, while a compressive load decreases the resonant frequency. As explained previously, there are two mechanisms responsible for frequency tuning; the stiffness from the arch curvature, and the axial stress. In our structures, the axial stress effect is dominant on the arch curvature. With structures A *(L* = 1000 μm) and C (*L* = 600 μm), the resonant frequency can be tuned down to −30% and −11%, respectively, of its initial value using a compressive load.

## 5. Conclusions

In this paper, we presented an experimental and simulation study of the tunability of in-plane arch-shaped clamped–guided beams. The first and third modes resonant frequencies were tuned by applying a bi-directional axial load for two types of structures using electrostatic actuation. It was found that a compressive axial load can have more effect for resonant frequency tuning than a tensile axial load. As the applied tensile load increases, the resonant frequency increases. For the compressive load, it was observed that the resonant frequency decreases. We conclude that the structure with four L-shaped beams holding the guided mass enables more tunability compared to the structure without the flexural beams. These four L-shaped beams help to have a symmetric *x*-displacement along the axial-load and prevent rotation. By applying tensile axial load, the resonant frequency of arch-shaped clamped–guided microresonators can be tuned up to 160% and 30% for first and third mode, respectively. With compressive axial load, the resonant frequency can be tuned down to −30% at low voltage loads. Additionally, we compared between the experimental and simulation results, which show good agreement. In conclusion, we have demonstrated that wide resonance frequency tuning is possible by employing a bidirectional electrostatic actuation scheme on arch-shaped clamped–guided resonators.

## Figures and Tables

**Table 1:** Geometrical dimensions of the resonator structures for Type 1 and Type 2.

**Type 1**	***L* (μm)**	***h* (μm)**	***b*_0_ (μm)**	***b* (μm)**	***g* (μm)**	***f*_01_ (kHz)**
A	1000	1.85	1.8	50	5	14.74
B	1000	1.85	2.6	50	5	14.87
C	600	1.85	1.8	50	2	42.7@30V
**Type 2**	***L* (μm)**	***h* (μm)**	***b*_0_ (μm**	***b* (μm)**	***g* (μm)**	***f*_01_ (kHz)**
D	1000	1.85	2.6	20	2	15
E	1000	1.85	2.6	10	2	14.2
F	1000	1.85	2.6	10	5	14.5
G	600	1.85	2.6	10	2	38.7

## References

[1] Zhang G., Gaspar J., Chu V., Conde J. (2005). Electrostatically actuated polymer microresonators. Appl. Phys. Lett..

[2] Arlett J., Myers E., Roukes M. (2011). Comparative advantages of mechanical biosensors. Nat. Nanotechnol..

[3] Li L. (2011). Recent development of micromachined biosensors. IEEE Sens. J..

[4] Eom K., Park H.S., Yoon D.S., Kwon T. (2011). Nanomechanical resonators and their applications in biological/chemical detection: Nanomechanics principles. Phys. Rep..

[5] Heinisch M., Reichel E., Dufour I., Jakoby B. (2012). Tunable resonators in the low kHz range for viscosity sensing. Sens. Actuators A Phys..

[6] Rhoads J.F., Shaw S.W., Turner K.L., Baskaran R. (2005). Tunable microelectromechanical filters that exploit parametric resonance. J. Vib. Acoust..

[7] Athukorala L., Budimir D. (2009). Compact dual-mode open loop microstrip resonators and filters. IEEE Microw. Wirel. Compon. Lett..

[8] Harne R., Wang K. (2013). A review of the recent research on vibration energy harvesting via bistable systems. Smart Mater. Struct..

[9] Al-Ashtari W., Hunstig M., Hemsel T., Sextro W. (2012). Frequency tuning of piezoelectric energy harvesters by magnetic force. Smart Mater. Struct..

[10] Hafiz M., Kosuru L., Younis M.I. (2016). Microelectromechanical reprogrammable logic device. Nat. Commun..

[11] Kozinsky I., Postma H.C., Bargatin I., Roukes M. (2006). Tuning nonlinearity, dynamic range, and frequency of nanomechanical resonators. Appl. Phys. Lett..

[12] Brueckner K., Niebelschuetz F., Tonisch K., Foerster C., Cimalla V., Stephan R., Pezoldt J., Stauden T., Ambacher O., Hein M. (2011). Micro-and nano-electromechanical resonators based on SiC and group III-nitrides for sensor applications. Phys. Status Solidi A.

[13] Rashvand K., Rezazadeh G., Mobki H., Ghayesh M.H. (2013). On the size-dependent behavior of a capacitive circular micro-plate considering the variable length-scale parameter. Int. J. Mech. Sci..

[14] Faroki H., Ghayesh M.H. (2015). Nonlinear dynamical behavior of geometrically imperfect microplates based on modified couple stress theory. Int. J. Mech. Sci..

[15] Ghayesh M.H., Faroki H., Alici G. (2015). Subcritical parametric dynamics of microbeams. Int. J. Eng. Sci..

[16] Huang X.M.H., Zorman C.A., Mehregany M., Roukes M.L. (2003). Nanoelectromechanical systems: Nanodevice motion at microwave frequencies. Nature.

[17] Lee J.E.-Y., Bahreyni B., Zhu Y., Seshia A.A. (2008). A single-crystal-silicon bulk-acoustic-mode microresonator oscillator. IEEE Electron Device Lett..

[18] Davila A.P., Jang J., Gupta A.K., Walter T., Aronson A., Bashir R. (2007). Microresonator mass sensors for detection of Bacillus anthracic Sterne spores in air and water. Biosens. Bioelectron..

[19] Ramos D., Arroyo-Hernandez M., Gil-Santos E., Duy Tong H., Van Rijn C., Calleja M., Tamayo J. (2009). Arrays of dual nanomechanical resonators for selective biological detection. Anal. Chem..

[20] Jun S.C., Huang X., Manolidis M., Zorman C., Mehregany M., Hone J. (2006). Electrothermal tuning of Al–SiC nanomechanical resonators. Nanotechnology.

[21] Remtema T., Lin L. (2001). Active frequency tuning for micro resonators by localized thermal stressing effects. Sens. Actuators A Phys..

[22] Sviličić B., Mastropaolo E., Zhang R., Cheung R. (2015). Tunable MEMS cantilever resonators electrothermally actuated and piezoelectrically sensed. Microelectron. Eng..

[23] Faroki H., Ghayesh M.H. (2015). Thermo-mechanical dynamics of perfect and imperfect Timoshenko microbeams. Int. J. Eng. Sci..

[24] Lee W.S., Kwon K.C., Kim B.K., Cho J.H., Youn S.K. (2004). Frequency-shifting analysis of electrostatically tunable micro-mechanical actuator. Comp. Model. Eng. Sci..

[25] Kafumbe S., Burdess J., Harris A. (2005). Frequency adjustment of microelectromechanical cantilevers using electrostatic pull down. J. Micromech. Microeng..

[26] Kao P.H., Dai C.L., Hsu C.C., Lee C.Y. (2009). Fabrication and characterization of a tunable in-plane resonator with low driving voltage. Sensors.

[27] Zhu D., Roberts S., Tudor M.J., Beeby S.P. (2010). Design and experimental characterization of a tunable vibration-based electromagnetic micro-generator. Sens. Actuators A Phys..

[28] Hajjaj A.Z., Alcheikh N., Ramini A., Al Hafiz M.A., Younis M.I. (2016). Highly tunable electrothermally and electrostatically actuated resonators. J. Microelectromech. Syst..

[29] Pourkamali S., Hashimura A., Abdolvand R., Ho G.K., Erbil A., Ayazi F. (2003). High-Q single crystal silicon HARPSS capacitive beam resonators with self-aligned sub-100-nm transduction gaps. J. Microelectromech. Syst..

[30] Krylov S., Ilic B.R., Schreiber D., Seretensky S., Craighead H. (2008). The pull-in behavior of electrostatically actuated bistable microstructures. J. Micromech. Microeng..

[31] Krylov S., Dick N. (2010). Dynamic stability of electrostatically actuated initially curved shallow micro beams. Contin. Mech. Thermodyn..

[32] Ouakad H.M., Younis M.I. (2010). The dynamic behavior of MEMS arch resonators actuated electrically. Int. J. Non-Linear Mech..

[33] Alkharabsheh S.A., Younis M.I. (2013). Statics and dynamics of mems arches under axial forces. J. Vib. Acoust..

[34] Abu-Salih S., Elata D. (2006). Experimental validation of electromechanical buckling. J. Microelectromech. Syst..

[35] Krylov S., Ilic B.R., Lulinsky S. (2011). Bistability of curved microbeams actuated by fringing electrostatic fields. Nonlinear Dyn..

[36] Ramini A., Alcheikh N., Ilyas S., Younis M.I. (2016). Efficient primary and parametric resonance excitation of bistable resonators. J. AIP Adv..

[37] MEMSCAP. http://www.memscap.com.

[38] Polytech. http://www.polytec.com/us.

